# Advanced Sensitive Feature Machine Learning for Aesthetic Evaluation Prediction of Industrial Products

**DOI:** 10.3390/jimaging12030131

**Published:** 2026-03-16

**Authors:** Jinyan Ouyang, Ziyuan Xi, Jianning Su, Shutao Zhang, Ying Hu, Aimin Zhou

**Affiliations:** School of Architecture and Art Design, Lanzhou University of Technology, Lanzhou 730050, China

**Keywords:** computer vision, aesthetic evaluation, machine learning, interpretability, optimization algorithm

## Abstract

As product aesthetics increasingly drive consumer preference, quantitative evaluation remains hindered by subjective evaluation biases and the black-box nature of modern artificial intelligence. This study proposes an advanced machine learning framework incorporating sensitivity-aware morphological features for the aesthetic evaluation of industrial products, with automotive design as a representative case. An aesthetic index system and its quantitative formulations are first developed to capture the morphological characteristics of product form. Subjective weights are determined via grey relational analysis (GRA), while objective weights are calculated using the coefficient of variation method (CVM) integrated with the technique for order preference by similarity to an ideal solution (TOPSIS). A game-theoretic weighting approach is then employed to fuse subjective and objective weights, thereby establishing a multi-scale aesthetic evaluation system. Sensitivity analysis is applied to identify six key indicators, forming a high-quality dataset. To enhance prediction performance, a novel model—improved lung performance-based optimization with backpropagation neural network (ILPOBP)—is proposed, where the optimization process leverages a maximin latin hypercube design (MLHD) to enhance exploration efficiency. The ILPOBP model effectively predicts aesthetic ratings based on limited morphological input data. Experimental results demonstrate that the ILPOBP model outperforms baseline models in terms of accuracy and robustness when handling complex aesthetic information, achieving a significantly lower test set mean absolute relative error (MARE = 4.106%). To further enhance model interpretability, Shapley additive explanations (SHAP) are employed to elucidate the internal decision-making mechanisms, offering reverse design insights for product optimization. The proposed framework offers a novel and effective approach for integrating machine learning into the aesthetic assessment of industrial product design.

## 1. Introduction

As modern industrial design and user experience become increasingly important, the aesthetic value of product forms has emerged as a key factor influencing user perception and purchase decisions [1,2,3]. Product form not only shapes the brand’s visual identity but also directly impacts consumers’ aesthetic preferences and purchasing behavior [4,5]. However, the aesthetic evaluation of product forms still primarily depends on the subjective judgment of designers, with a lack of systematic and scientifically grounded quantitative methods [6]. Furthermore, aesthetic evaluation is inherently subjective and complex, with significant variations in how different users perceive the aesthetics of the same product, adding uncertainty to design optimization. Therefore, finding ways to combine both subjective and objective evaluation methods to scientifically and accurately quantify the aesthetic value of products has become a critical issue in contemporary design research [7,8].

Traditional aesthetic research emphasizes qualitative analysis, inductive reasoning, and philosophical speculation. In contrast, computational aesthetics draws on theories from cognitive science, computational science, and experimental psychology to quantitatively describe aesthetic knowledge. It grounds aesthetic evaluation in validated knowledge, collective experience, and logical inference, offering a new methodological and technical approach to aesthetic research [9,10]. Currently, there are two primary methods for aesthetic evaluation of product forms: subjective and objective evaluation. Subjective evaluation relies on survey methods to collect data or assigns subjective weights through expert interviews to construct an evaluation model [11]. Common survey methods include questionnaires, Likert scales, and the Semantic Differential Method (SD) [12,13,14]. In contrast, objective evaluation is based on the principles and methods of computational aesthetics, constructing an aesthetic evaluation system for product forms and quantitatively describing aesthetic indicators to build an evaluation model [15]. These two methods reflect different aspects of aesthetic value, each with its own advantages and limitations. Subjective evaluation captures the diversity of individual aesthetic perception, while objective evaluation provides a rational basis for aesthetic analysis. However, no single method can fully capture the complex, multidimensional interactions inherent in aesthetic evaluation. Therefore, there is an urgent need to develop a comprehensive evaluation system that integrates both subjective and objective information to improve the systematization and comprehensiveness of aesthetic evaluation. Furthermore, recent cross-disciplinary research has demonstrated that integrating advanced mathematical frameworks can significantly enhance complex multi-attribute decision-making under uncertainty [16,17].

Traditional methods like Grey Relational Analysis (GRA), Principal Component Analysis (PCA), the Entropy Weight Method, and the Technique for Order Preference by Similarity to an Ideal Solution (TOPSIS) have been widely applied in product form aesthetic evaluation [18,19,20,21,22]. These methods are characterized by clear computational principles, ease of use, and simplicity in implementation. However, they also have significant limitations, such as insufficient information utilization, high data requirements, sensitivity to extreme values, and susceptibility to bias. To overcome similar limitations in managing subjective ambiguity and imprecise data, recent studies have successfully employed advanced topological models and intuitionistic fuzzy rough graphs in various complex environments [23,24]. Inspired by these approaches, managing the inherent subjective ambiguity in product aesthetic evaluation requires equally robust integrated methods.

In recent years, with the rapid development of big data and artificial intelligence technologies, researchers have explored aesthetic evaluation using models such as Linear Regression (LR), Support Vector Machines (SVM), Decision Trees (DT), Random Forests (RF), and Convolutional Neural Networks (CNN) [25,26,27,28,29]. Although these methods have shown promising results, they still face challenges in practical applications, including the need for improved prediction accuracy, incomplete aesthetic indicator systems, low model reliability, and poor interpretability.

This study aims to address the following core issues: (1) How to effectively combine subjective and objective aesthetic evaluations to improve the reliability and validity of the assessment. (2) Can machine learning methods be used to improve the accuracy of product form aesthetic evaluations and better support the optimization of product form design?

To address these challenges, this study proposes a highly domain-specific, synergistic framework that integrates multiple methodologies to resolve the unique bottlenecks of industrial design evaluation. First, to rigorously resolve the inherent subjective-objective duality of aesthetics, Grey Relational Analysis (GRA) and the CVM-TOPSIS method are fused via game theory. This specific combination effectively balances human psychological preferences with physical geometric variances, generating highly reliable comprehensive aesthetic labels. Next, to prevent the curse of dimensionality and overfitting, correlation-based sensitivity analysis bridges the gap by distilling 17 initial indicators into 6 core features. This lightweight, semantic-rich input space perfectly synergizes with our proposed MLHD-LPO optimized Back Propagation Neural Network (BPNN), ensuring robust convergence and high predictive accuracy. Finally, breaking away from traditional black-box models, the integration of Shapley Additive Explanations (SHAP) provides explicit structural interpretability. It translates neural network outputs into feature-level diagnostic feedback, shifting the paradigm from merely evaluating aesthetics to actively guiding actionable form optimization. Ultimately, the core novelty of this study lies in this methodological synergy, which transforms abstract aesthetic theories into a computable, interpretable engineering workflow, laying the algorithmic foundation for intelligent computer-aided design platforms.

## 2. Dataset Construction and Processing

### 2.1. Development of the Aesthetic Indicator System for the Car Front Face

In product form design and aesthetic evaluation, the principles of formal aesthetics and Gestalt psychology serve as widely applied theoretical foundations. These principles represent experiential summaries and generalizations of human aesthetic practice and the associative rules that have evolved in human aesthetic thinking [30,31]. Based on these principles, this study develops an aesthetic evaluation indicator system for product forms, consisting of 17 indicators, including balance, proportion, rhythm, order, unity, symmetry, regularity, common direction (parallelism), continuity, simplification, similarity, similar proportion, attractiveness, stability, hierarchy, contrast, and complexity. The detailed indicators are listed in Appendix A.

**Figure 1:**
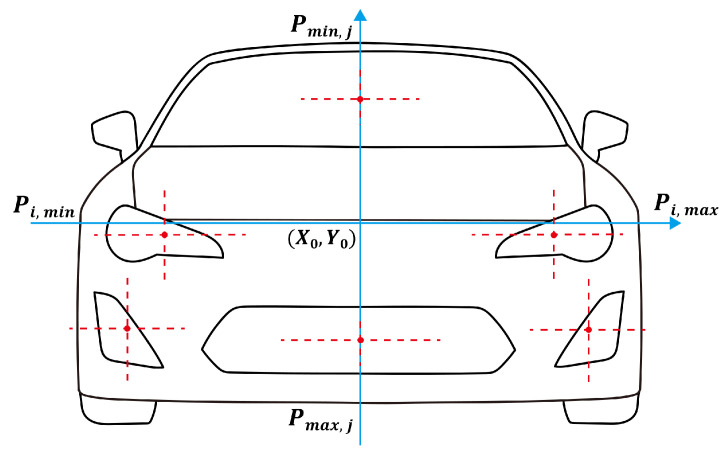
Coordinate system of the car front face.

For example, the coordinate system for calculating aesthetic indicators of the car front face is shown in Figure 1, with the origin located at the center of the product’s outline. To eliminate scale effects on evaluation results, the width of all samples is standardized to a uniform value for subsequent calculations. Based on this, a formula for calculating the aesthetic indicators of the car front face is constructed, drawing on previous research [20,32].

### 2.2. Construction of the Comprehensive Aesthetic Evaluation Model Using Subjective and Objective Information

In product form design, aesthetic evaluation plays a central role in the decision-making process, encompassing a range of multidimensional and complex features. This process begins with the visual perception of light reflected from the product surface and captured by the eyes. The reflected light signals are then converted into bioelectrical signals, which are processed by the brain. In the brain, these signals undergo a series of complex cognitive steps, including retrieval, extraction, reorganization, and synthesis of information, ultimately resulting in an organized and structured aesthetic perception. This process is influenced by an individual’s experiences, cultural background, and personal preferences, which leads to significant variations in aesthetic evaluations of the same product form. This highlights that aesthetic cognition integrates both sensibility and rationality, and that aesthetic evaluation is a blend of both subjectivity and objectivity. Therefore, we combine subjective and objective evaluation information to conduct an aesthetic evaluation of the car front face form.

**Figure 2:**
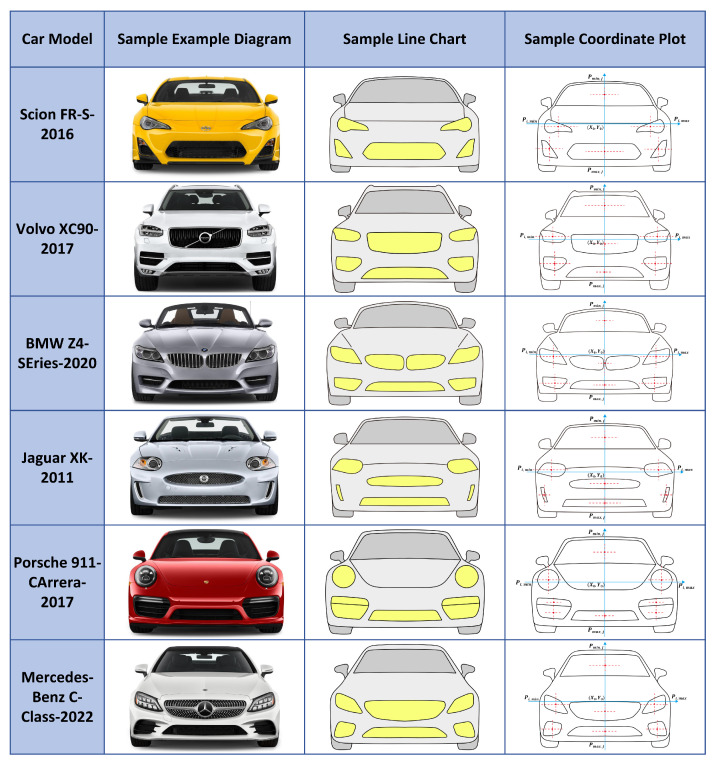
Six randomly selected car front face samples.

In this study, we collected the necessary high-definition images of car front faces and corresponding subjective aesthetic rating data from the professional automotive review website, The Car Connection. Unlike ad hoc laboratory surveys, the styling and aesthetic scores provided by this platform are aggregated consensus ratings formulated by panels of seasoned automotive journalists and industry experts based on standardized evaluation criteria. To ensure the data’s timeliness and representativeness, we selected car models from the most recent release years. A total of 400 high-definition images of car front faces were collected and standardized for analysis and evaluation. These images and their corresponding expert ratings serve as the foundational computational samples for our study. A portion of the sample is shown in Figure 2.

#### 2.2.1. Subjective Evaluation Based on Grey Relational Analysis

Subjective evaluation primarily relies on the basic data collected through user surveys and expert interviews to construct the evaluation model. Because subjective aesthetic perceptions inherently contain ambiguity and human cognitive uncertainty, Grey Relational Analysis (GRA) is specifically chosen as an effective tool for handling such uncertain information. GRA describes the relationships between various factors by calculating the grey relational degree of each factor in the sample data [33]. This method uses similarity or dissimilarity to assess the interactions between factors, and thus mathematically measures the strength of their association without requiring massive sample sizes. In the subjective aesthetic evaluation process, we use GRA to calculate the correlation between subjective aesthetic evaluation values and aesthetic indicators, thereby accurately determining the subjective weights of each aesthetic indicator. The detailed implementation steps are as follows:

Let the number of product form samples and aesthetic indicators be denoted by *n* and *m*, respectively. The aesthetic evaluation values of the samples are used as the reference sequence:

(1) $$X_{0}=\left(x_{0}\left(1\right),x_{0}\left(2\right),\ldots ,x_{0}\left(n\right)\right)$$

The aesthetic indicator values of each sample are taken as the comparison sequences:

(2) $$X_{1}=\left(x_{1}\left(1\right),x_{1}\left(2\right),\ldots ,x_{1}\left(n\right)\right)$$

$$X_{i}=\left(x_{i}\left(1\right),x_{i}\left(2\right),\ldots ,x_{i}\left(n\right)\right)$$

$$X_{m}=\left(x_{m}\left(1\right),x_{m}\left(2\right),\ldots ,x_{m}\left(n\right)\right)$$

The absolute differences, maximum, and minimum absolute differences between the two sequences are as follows:

(3) $$\Delta_{0i}\left(k\right)=\left|x_{0}\left(k\right)-x_{i}\left(k\right)\right|$$

(4) $$\Delta_{\min}=\min_{i}\min_{k}\left|x_{0}\left(k\right)-x_{i}\left(k\right)\right|$$

(5) $$\Delta_{\max}=\max_{i}\max_{k}\left|x_{0}\left(k\right)-x_{i}\left(k\right)\right|$$

The grey relational coefficient of $X_{0}$ to $X_{i}$ at the *k*-th sample is:

(6) $$\tau \left(x_{0}\left(k\right),x_{i}\left(k\right)\right)=\frac{\Delta_{\min}+\rho \Delta_{\max}}{\Delta_{0i}\left(k\right)+\rho \Delta_{\max}}$$

where ρ∈(0,1) is the distinguishing coefficient, which primarily adjusts the degree of contrast between aesthetic indicators. Its value significantly influences the calculation results. In this study, we set it to 0.5. The grey relational coefficient of the *j*-th aesthetic indicator is:

(7) $$r_{j}=\frac{1}{n}\sum_{k=1}^{n}\tau \left(x_{0}\left(k\right),x_{i}\left(k\right)\right)$$

After normalization, the subjective weight of each indicator is:

(8) $$\omega_{j}=\frac{r_{j}}{\sum_{j=1}^{m}r_{j}}$$

#### 2.2.2. Objective Evaluation Using the Coefficient of Variation-TOPSIS Method

To objectively quantify the geometric and structural features of product forms without human bias, the TOPSIS method is selected. TOPSIS is a robust multi-criteria decision-making method that performs comprehensive evaluation by comparing the distance between sample values and ideal values [34]. This method uses relative closeness to characterize the distance between each evaluation object and reference points. In the implementation of TOPSIS, reference points are first determined in the decision space, including the optimal (ideal) point and the worst (anti-ideal) point. Then, the distances between each evaluation object and the reference points are calculated. An evaluation object is considered better if it is closer to the ideal point or further from the anti-ideal point.

Although traditional TOPSIS models typically use the average weighting method to determine weights, this approach may not accurately reflect the actual importance of different indicators in decision-making, as it neglects the differences among indicators. Therefore, this study integrates the Coefficient of Variation Method (CVM) into the TOPSIS model to determine objective weights [35,36]. This approach dynamically assigns weights based on the variation in the evaluated indicators, capturing differences in their importance more effectively. The detailed implementation steps are as follows:

Let the weight of the aesthetic indicator be $\omega_{j}$, and let $x_{i}{\left(k\right)}^{\prime }$ represent the normalized data after positive transformation. The weighted data $r_{i}\left(k\right)$ can be calculated as:

(9) $$r_{i}\left(k\right)=\omega_{j}x_{i}{\left(k\right)}^{\prime }$$

The distances between the processed data and the extreme values are then calculated. After processing, the data matrix can be formed as:

(10) $$R={\left(r_{i}\left(k\right)\right)}_{m\times n}$$

Define the maximum value of each indicator (column) as $r_{j}^{+}$:

(11) $$r_{j}^{+}=\max\left(r_{1}\left(k\right),r_{2}\left(k\right),\ldots ,r_{m}\left(k\right)\right)$$

The distance of the *i*-th evaluation object from the maximum value is defined as $d_{i}^{+}$:

(12) $$d_{i}^{+}=\sqrt{\sum_{k=1}^{n}{\left(r_{j}^{+}-r_{i}\left(k\right)\right)}^{2}}$$

The distance of the *i*-th evaluation object from the minimum value is defined as $d_{i}^{-}$:

(13) $$d_{i}^{-}=\sqrt{\sum_{k=1}^{n}{\left(r_{j}^{-}-r_{i}\left(k\right)\right)}^{2}}$$

The comprehensive evaluation score is then calculated as:

(14) $${\mathrm{Score}}_{i}=\frac{d_{i}^{-}}{d_{i}^{+}+d_{i}^{-}}$$

#### 2.2.3. Integrated Subjective and Objective Evaluation Using the Game Theory-Based Combination Weighting Method

Considering the dual subjective and objective nature of aesthetic evaluation, this study employs a game theory-based combination weighting method to integrate subjective and objective weight information [37]. This approach constructs a comprehensive evaluation model that accounts for both perspectives. Game theory, as a mathematical theory that studies competitive interaction phenomena, focuses on analyzing rational behaviors and decision-making processes in multi-party games [38]. Through this combination weighting method, subjective and objective weights are balanced and reconciled, ensuring that the fusion process retains as much information as possible from both sources. The final result calculates the comprehensive weights of aesthetic indicators, reducing the bias and limitations of relying solely on either subjective or objective evaluation methods. This enhances the objectivity, reliability, and practical relevance of the aesthetic evaluation results. The specific steps are as follows:

Establishing the basic weight vector set:

Define $W_{q}=\left\{\omega_{1},\omega_{2},\ldots ,\omega_{m}\right\}\;\left(q=1,2,\ldots ,p\right)$, where *W* represents the weight set determined by the *p*-th weighting method, *m* is the number of aesthetic indicators, and *p* is the number of weighting methods. In this study, p=2. Let $\alpha =\{\alpha_{1},\alpha_{2}\}$ be the set of linear combination coefficients. The linear combination of the two weight vectors is expressed as:

(15) $$W_{1}=\alpha_{1}\omega_{1}^{⊤}+\alpha_{2}\omega_{2}^{⊤}$$

Optimization of the linear combination coefficients:

Based on the concept of the game aggregation model, the deviation minimization is set as the goal to optimize the two linear combination coefficients, resulting in the most satisfactory weights in $W_{1}$. The objective function is formulated as:

(16) $$\min{∥\sum_{p=1}^{n}\alpha_{p}\omega_{p}^{⊤}-\omega ∥}_{2}$$

Transform the above equation into an equivalent linear system by applying the first-order derivative condition for optimization:

(17) $$\left[\begin{array}{cc} \omega_{1}\omega_{1}^{⊤} & \omega_{1}\omega_{2}^{⊤}\\ \omega_{2}\omega_{1}^{⊤} & \omega_{2}\omega_{2}^{⊤} \end{array}\right]\left[\begin{array}{c} \alpha_{1}\\ \alpha_{2} \end{array}\right]=\left[\begin{array}{c} \omega_{1}\omega_{1}^{⊤}\\ \omega_{2}\omega_{2}^{⊤} \end{array}\right]$$

Normalization of the optimization coefficients:

Compute the optimized combination coefficients $\alpha_{1}$ and $\alpha_{2}$, followed by normalization:

(18) $$\left\{\begin{array}{c} \alpha_{1}^{*}=\alpha_{1}/\left(\alpha_{1}+\alpha_{2}\right)\\ \alpha_{2}^{*}=\alpha_{2}/\left(\alpha_{1}+\alpha_{2}\right) \end{array}\right.$$

Calculation of the comprehensive weights:

Finally, obtain the comprehensive weights of the aesthetic indicators:

(19) $$W_{1}=\alpha_{1}^{*}\omega_{1}^{⊤}+\alpha_{2}^{*}\omega_{2}^{⊤}$$

### 2.3. Sensitivity Analysis of Morphological Aesthetic Features

This study applies relevant theories to the quantitative evaluation of the aesthetic value of automobile front faces. Based on the analyses in Section 2.1 and Section 2.2, 17 aesthetic indicators influencing the aesthetic appeal of automobile front faces and their comprehensive aesthetic values were identified. However, due to the complexity and large scale of the evaluation system, existing methods struggle to achieve efficient and lightweight evaluations of automobile front face aesthetics. This limitation conflicts with the study’s objective of addressing the issue efficiently. To resolve this, sensitivity analysis was conducted on the collected data to assess the relationship between each aesthetic indicator and the comprehensive aesthetic value. Specifically, the Pearson Correlation Coefficient was employed to evaluate the linear correlation between individual aesthetic indicators and the comprehensive aesthetic value, identifying key aesthetic indicators.

To determine the final subset of features, a strict selection criterion was established: only the top-ranking indicators demonstrating a significant positive driving effect on the comprehensive aesthetic score (specifically, a positive correlation coefficient r>0.2) were retained. Consequently, six key aesthetic indicators—Symmetry, Regularity, Common directionality, Similarity, Attractiveness, and Proportional similarity—were identified as the most sensitive features. Indicators exhibiting weak (near-zero) or negative correlations were intentionally excluded to prevent the introduction of noise and to mitigate the risk of the “curse of dimensionality”. By extracting these six core features, the study provides critical and lightweight input features for subsequent applications of machine learning methods, thereby significantly improving the computational efficiency, interpretability, and practicality of the model.

The Pearson Correlation Coefficient is a widely used method for assessing the strength and direction of the linear relationship between two variables [39]. It is particularly suitable for analyzing the multidimensional characteristics of aesthetic indicators and their influence on comprehensive aesthetic values. By quantifying the correlation between each aesthetic indicator and the comprehensive aesthetic value, the key aesthetic indicators with significant impact on aesthetic evaluation can be identified. The correlation is calculated using the following formula:

(20) $$r=\frac{\sum_{i=1}^{n}\left(X_{i}-\bar{X}\right)\left(Y_{i}-\bar{Y}\right)}{\sqrt{\sum_{i=1}^{n}{\left(X_{i}-\bar{X}\right)}^{2}}\sqrt{\sum_{i=1}^{n}{\left(Y_{i}-\bar{Y}\right)}^{2}}}$$

where *r* represents the Pearson Correlation Coefficient; *n* is the total number of data points; $X_{i}$ and $Y_{i}$ are the values of each paired observation; $\bar{X}$ is the mean of all observations of *X*; $\bar{Y}$ is the mean of all observations of *Y*. The numerator represents the sum of the product of *X* and *Y* deviations from their respective means, reflecting the degree of their covariation. The denominator is the Euclidean norm of the deviations of *X* and *Y* from their respective means. This denominator acts as a normalization factor, ensuring that the value of *r* lies between −1 and 1. The sign of *r* indicates the direction of the relationship (positive or negative correlation), while the magnitude of its absolute value indicates the strength of the correlation.

**Figure 3:**
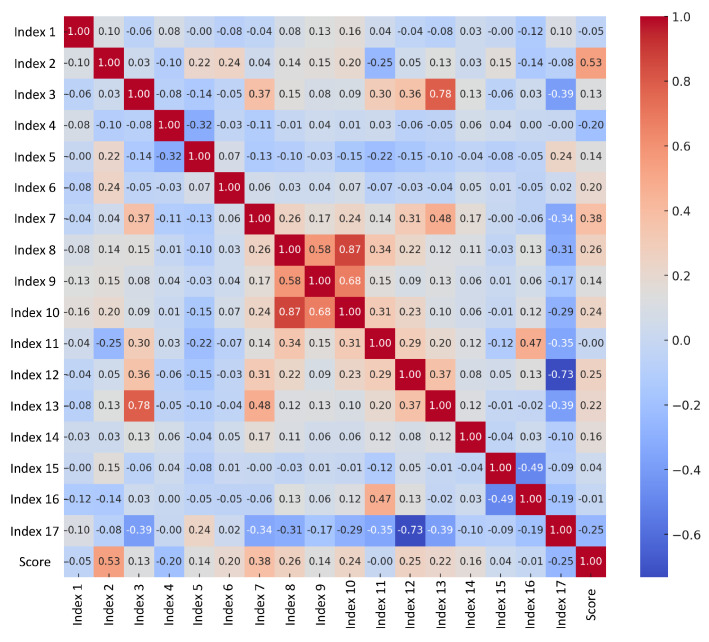
Heatmap of correlations among 17 aesthetic indicators.

Figure 3 presents the correlation heatmap of the 17 aesthetic indicators, and Table 1 provides the specific correlation coefficients. Analyzing the correlation coefficients revealed that six aesthetic indicators—symmetry, regularity, common direction (parallelism), similarity, attractiveness, and proportional similarity—exhibit significant linear relationships with the comprehensive aesthetic value of automobile front faces. By performing the above steps, the complexity of the evaluation model is reduced, effectively enhancing analysis efficiency. This is a critical step in enabling machine learning models to predict comprehensive aesthetic values accurately.

**Figure 4:**
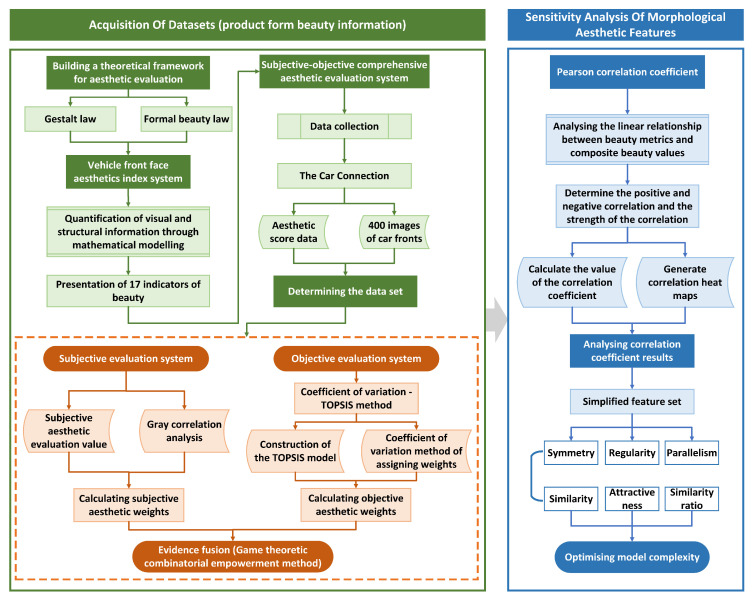
Flowchart for acquiring the dataset.

Through the complete steps outlined in Section 2.1, Section 2.2 and Section 2.3, we established a robust system of aesthetic indicators and a comprehensive subjective and objective evaluation framework. Sensitivity analysis further allowed us to construct a complete training dataset. Figure 4 illustrates the workflow for obtaining the dataset.

## 3. Machine Learning Model

To address the high-dimensional characteristics and complexity of aesthetic indicators and evaluation data, this study leverages the capabilities of machine learning to develop a machine learning-based aesthetic evaluation model. To overcome the limitations of traditional BP neural networks in handling high-dimensional data, the MLHD strategy is introduced. This strategy achieves a balance between uniform sampling and global exploration during the parameter initialization phase, enhancing the model’s generalization ability. Additionally, the BP neural network is further optimized and improved using the LPO algorithm, aiming to enhance the predictive performance of the model.

### 3.1. Improved Lung Performance-Based Optimization Algorithm

#### 3.1.1. Lungs Performance-Based Optimization

The LPO algorithm is a novel bio-inspired optimization algorithm whose core concept is to simulate the gas exchange process in the lungs to optimize the parameters of computational models [40]. Key processes in the LPO model include gas intake and release, carbon dioxide separation, and blood circulation in veins. These processes are mapped to specific operational steps within the optimization algorithm. The objective of the algorithm is to maximize oxygen absorption while minimizing energy consumption. In our target task, the algorithm is used to identify the optimal parameters of the BP neural network, such as weights and biases, to minimize the error (or loss) function. The detailed steps of the LPO algorithm are as follows:

1. Initialization:

At the beginning of the algorithm, a population or air quality swarm ($M_{i}$, where $i=1,2,\ldots ,N_{\mathrm{pop}}$) is initialized. This initial population is randomly generated within the problem’s target range, bounded by a maximum value $M_{\max}$ and a minimum value $M_{\min}$.

2. Lung function modeling:

Lung functionality is modeled as an electrical circuit, specifically an RC circuit (resistor-capacitor circuit). The real part of the impedance represents respiratory resistance ($Z_{R}$), while the imaginary part represents respiratory reactance ($Z_{X}$). The volume of gas diffusing through tissues follows Fick’s law:

(21) $$V=\frac{AK\Delta P}{d}$$

where *A* is the cross-sectional area, *d* is the tissue thickness, *K* is the gas diffusion coefficient, and ΔP is the pressure gradient.

The pressure variation inside the lungs is given by:

(22) $$\Delta P=V\times \sqrt{R^{2}+{\left(\frac{1}{2\pi f_{r}RC}\right)}^{2}}\times \sin\left(2\pi f_{r}t\right)\times \sin\left(2\pi f_{r}t+\theta \right)$$

where $f_{r}$ is the breathing frequency, *t* is time, *R* is resistance, and *C* is capacitance.

3. Population movement (Inhalation and Exhalation):

Inhalation: The position of the initial population is updated using the formula:

(23) $$\begin{array}{cc} M_{\mathrm{new},1}^{i} & =M_{i}+M_{i}\times \sqrt{R_{i}^{2}+{\left(\frac{1}{2\pi f_{r}R_{i}C_{i}}\right)}^{2}}\\ \; & \;\times \sin\left(2\pi f_{r}t\right)\times \sin\left(2\pi f_{r}t+\theta_{i}\right) \end{array}$$

This equation simulates the process of air masses (population members) entering the lungs (problem space). Exhalation: Oxygen is separated from the air and enters the bloodstream, modeled as:

(24) $$\begin{array}{cc} M_{\mathrm{new},2}^{i}= & M_{\mathrm{new},1}^{i}+K_{12}\times \alpha_{i}\times \left(M_{\mathrm{new},1}^{i}-M_{1}\right)\\ & \;+K_{23}\times \alpha_{i}\times \left(M_{3}-M_{2}\right) \end{array}$$

Here, $K_{ij}$ determines the direction of movement based on fitness values, and $\alpha_{i}$ represents the displacement toward a better solution.

Carbon Dioxide Separation (Population combination and crossover): For each dimension *j*, if $s_{i}>\mathrm{rand}$, the position is updated as:

(25) $$m_{\mathrm{new},3}^{ij}=m_{1}^{j}+\alpha_{i}\times \left(m_{3}^{j}-m_{2}^{j}\right)$$

Otherwise, the position remains unchanged:

(26) $$m_{\mathrm{new},3}^{ij}=m_{\mathrm{new},2}^{ij}$$

During this process, $s_{i}$ is a probability value that decreases with each exhalation and is inversely proportional to the number of inhalation and exhalation cycles $N_{e}$:

(27) $$s_{i}=\frac{\mathrm{rand}}{N_{e}}$$

If the position $M_{\mathrm{new},3}^{ij}$ improves the objective function, it replaces the current position.

4. Iteration:

The optimization loop continues for a predefined number of iterations or pulses, with the goal of improving the objective function at each step.

5. Computational complexity:

The computational complexity is determined by the population size $N_{\mathrm{pop}}$, problem dimensions *D*, and maximum iterations ${\mathrm{Iter}}_{\max}$. The overall complexity is expressed as:

(28) $$\begin{array}{cc} O(\mathrm{LPO}) & =O\left(\mathrm{Cost}\right)\times (O\left(N_{\mathrm{pop}}\right)\\ \; & \;+2\times O(N_{\mathrm{pop}}\times {\mathrm{Iter}}_{\max}\times N_{e}\times D)) \end{array}$$

The LPO algorithm demonstrates exceptional performance in various optimization problems by simulating these natural processes. However, the standard LPO has certain limitations in practical applications. For instance, the random generation of the initial population may result in insufficient diversity of search starting points, which can negatively affect the algorithm’s global search capability. To address this issue, this study proposes an improved LPO algorithm based on the MLHD, enhancing the quality of the initialization phase in the standard LPO.

#### 3.1.2. Maximin Latin Hypercube Design Initialization

To enhance the quality of the initial population in the LPO algorithm, this study employs the MLHD strategy for population initialization [41]. This strategy ensures that individuals are uniformly and diversely distributed in the solution space at the initialization stage, improving the algorithm’s global exploration capability and increasing its likelihood of escaping local optima. The MLHD initialization process consists of the following steps:

1. Partitioning the design space:

First, determine the population size *N* and the dimensionality of the population *D*. In each dimension, the design space is divided into *N* equal subintervals, constructing a *D*-dimensional grid structure. Each subinterval can be occupied by only one sample point, ensuring uniform distribution along each dimension and maintaining diversity in the population.

2. Determining sample point positions:

For each dimension *d* (d=1,…,D), randomly select a point within each subinterval as the sample position. The sample point $x_{d,n}$ for the *n*-th subinterval (n=1,…,N) in dimension *d* is determined using the following formula:

(29) $$x_{d,n}=a_{d}+\left(n-\frac{1}{2}+\epsilon_{d,n}\right)\frac{\left(b_{d}-a_{d}\right)}{N}$$

Here, $a_{d}$ and $b_{d}$ are the lower and upper bounds of dimension *d*, respectively, and $\epsilon_{d,n}$ is a random value generated within the interval [−0.5,0.5]. This random component increases the randomness of sample points within subintervals, ensuring that the sample points are as dispersed as possible.

3. Maximizing the minimum distance between points:

To ensure a uniform distribution of sample points across the entire design space, an iterative optimization method is applied to adjust the sample point positions, maximizing the minimum distance between points. The optimization objective is described as:

(30) $$\max_{X}\left(\min_{i\neq j}d\left(x_{i},x_{j}\right)\right)$$

where $d(x_{i},x_{j})$ represents the Euclidean distance between sample points $x_{i}$ and $x_{j}$, calculated as:

(31) $$d\left(x_{i},x_{j}\right)={∥x_{i}-x_{j}∥}_{2}=\sqrt{\sum_{k=1}^{D}{\left(x_{i,k}-x_{j,k}\right)}^{2}}$$

In this equation, X denotes the set of all sample points $\left\{x_{1},x_{2},\ldots ,x_{N}\right\}$, and $x_{i}$ and $x_{j}$ are two sample points in the *D*-dimensional space. The goal of the optimization is to find a configuration of sample points such that the minimum distance between any two points in the set is as large as possible, thereby ensuring a balanced distribution of the population.

**Figure 5:**
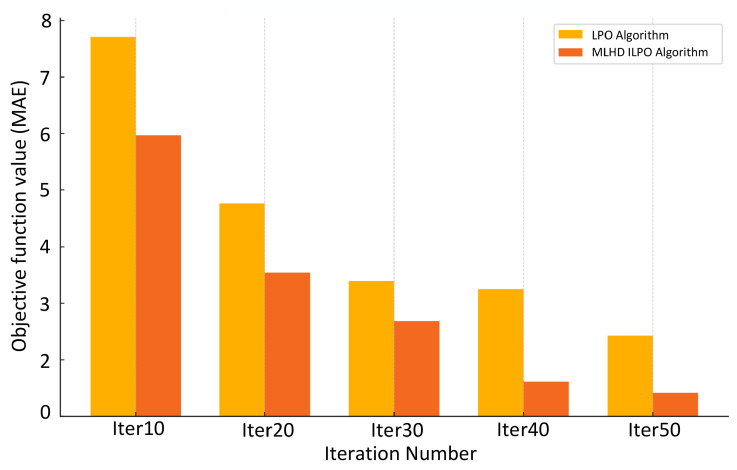
Optimization performance comparison between LPO algorithm and MLHD-enhanced LPO algorithm with different number of iterations.

A comparison of the optimization performance between the LPO algorithm and the MLHD-enhanced LPO algorithm at different iteration numbers is shown in Figure 5. From the results, it can be seen that the MLHD-LPO algorithm outperforms the traditional LPO algorithm at all key iteration points, especially at the later iterations (40th and 50th), the performance improvement is more significant, which verifies the positive impact of the MLHD initialization mechanism on the convergence of the algorithm.

### 3.2. Improved LPO Algorithm for Optimizing a BP Neural Network (ILPOBP)

In Section 3.1.2, we proposed an improved LPO algorithm based on the MLHD to enhance the performance of the standard LPO algorithm in global optimization problems. This section details how the improved LPO algorithm is applied to the optimization of BP neural networks, particularly in adjusting the network’s weights and bias parameters.

**Figure 6:**
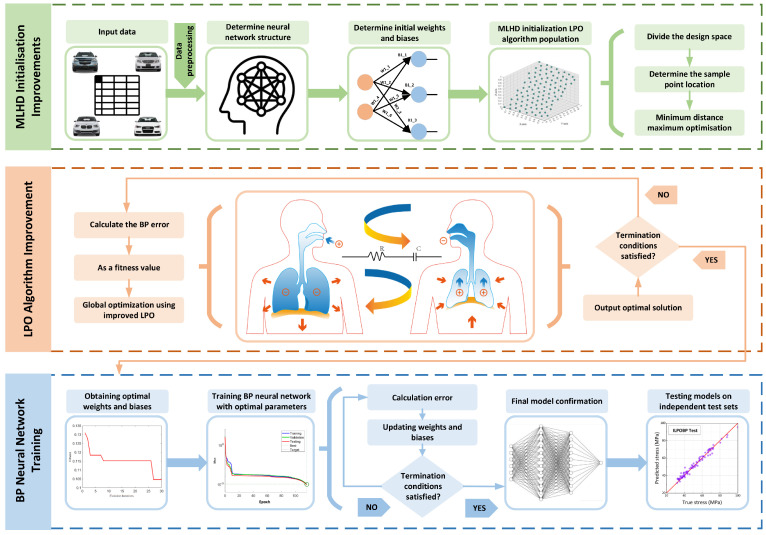
Flowchart of the improved LPO algorithm optimizing the BP neural network (ILPOBP).

The BP neural network is a powerful nonlinear model, but its performance is highly dependent on the selection of network parameters [42]. Traditional gradient descent methods often face challenges such as falling into local optima and being sensitive to initialization choices, which can adversely affect the model’s convergence and performance. To address these issues, this study utilizes the improved LPO algorithm to optimize the weights and biases of the BP neural network, thereby enhancing its predictive accuracy and generalization ability. As shown in Figure 6, the steps for optimizing the BP neural network with the improved LPO algorithm are as follows:

1. Network architecture definition: First, define the architecture of the BP neural network, including the number of neurons in the input layer, one or more hidden layers, and the output layer. The specific structure of the network is determined based on the requirements of the application scenario.
2. Parameter initialization: The initial weights and bias parameters of the BP neural network are generated using the MLHD initialization strategy. MLHD ensures that the initial parameters are uniformly distributed across the entire search space, enhancing the global search capability of the LPO algorithm and reducing the risk of falling into local optima.
3. Fitness function definition: The fitness function is defined as the loss function of the BP neural network, specifically the mean squared error (MSE). During the optimization process, the fitness function evaluates the performance of each individual (i.e., a set of network parameters) in the LPO algorithm.
4. ILPO algorithm optimization: Apply the improved LPO (ILPO) algorithm for global optimization. The LPO algorithm simulates the inhalation and exhalation processes of lungs to update the population’s positions. In each iteration, the network parameters (e.g., weights and biases) are updated based on the inhalation and exhalation phases of the LPO algorithm:
  Inhalation phase: Explores new solution spaces by simulating air entering the lungs. Position updates follow the inhalation formula detailed in Section 3.1.1. Exhalation phase: Further optimizes and adjusts the new solutions by simulating the oxygen exchange process, gradually eliminating suboptimal solutions and retaining individuals with higher fitness.
  Through population combination and crossover operations, the diversity of the population is ensured, progressively converging toward the optimal solution.
5. Network training: Train the BP neural network using the parameter set (weights and biases) optimized by the ILPO algorithm. The LPO-optimized parameters significantly improve the network’s convergence speed and overall performance.
6. Performance evaluation: Evaluate the performance of the ILPO-optimized BP neural network on the validation set, using metrics such as accuracy and mean squared error. If the performance does not meet expectations, return to Step 4 to continue optimization.
7. Iterative updates: Based on the performance evaluation results, if the network performance does not meet the desired target, continue updating the network parameters through ILPO optimization until the performance criteria are satisfied or the maximum number of iterations is reached.
8. Final model confirmation: Select the parameter set that performs best on the validation set as the parameters for the final BP neural network model.
9. Model testing: Test the final model’s performance on an independent test set to evaluate its generalization ability.

The improved LPO algorithm (ILPO) effectively enhances the performance of the BP neural network through the following mechanisms:

1. Increased diversity of the initial population: By employing the MLHD strategy to generate the initial population, ILPO ensures a uniform distribution of individuals across the solution space. This enhances the global search capability of the LPO algorithm.
2. Combination of global search and local optimization: The inhalation and exhalation processes in the LPO algorithm simulate a balance between exploration and exploitation. This enables the algorithm to maintain a good trade-off between global search and local convergence, effectively avoiding entrapment in local optima.
3. Fitness-Driven adaptive updates: By simulating the gas exchange process within biological systems, the LPO algorithm adaptively adjusts the search trajectory, improving the efficiency and accuracy of convergence.

## 4. Results

### 4.1. Performance Evaluation of the ILPOBP Model

In this study, the improved ILPOBP model was employed to analyze and predict the “comprehensive aesthetic value” of automobile front faces. To comprehensively evaluate the model’s effectiveness in product aesthetic prediction, four key statistical metrics were used as evaluation criteria.

1. Mean Squared Error (MSE):

MSE measures the average squared difference between predicted values and actual values, reflecting the model’s predictive accuracy. It is particularly sensitive to outliers. Lower MSE values indicate better model performance. The formula is:

(32) $$\mathrm{MSE}=\frac{1}{n}\sum_{i=1}^{n}{\left(y_{i}-{\hat{y}}_{i}\right)}^{2}$$

where $y_{i}$ is the actual value, ${\hat{y}}_{i}$ is the predicted value, and *n* is the total number of predictions.

2. Root Mean Squared Error (RMSE):

RMSE is the square root of MSE and provides a more interpretable metric consistent with the unit of the target variable. It emphasizes larger prediction errors and reflects the magnitude of the average error. The formula is:

(33) $$\mathrm{RMSE}=\sqrt{\frac{1}{n}\sum_{i=1}^{n}{\left(y_{i}-{\hat{y}}_{i}\right)}^{2}}$$

Similarly to MSE, lower RMSE values indicate better model performance.

3. Mean Absolute Error (MAE):

MAE measures the average absolute difference between predicted values and actual values. Unlike MSE and RMSE, MAE is less sensitive to outliers and provides a realistic representation of the model’s average prediction error across the dataset. The formula is:

(34) $$\mathrm{MAE}=\frac{1}{n}\sum_{i=1}^{n}\left|y_{i}-{\hat{y}}_{i}\right|$$

where $y_{i}$ is the actual value, ${\hat{y}}_{i}$ is the predicted value, and *n* is the total number of predictions. Lower MAE values indicate smaller average prediction errors and better performance.

4. Mean Absolute Relative Error (MARE):

MARE quantifies the average relative error between predicted and actual values, expressed as a percentage. It is particularly useful for comparing errors across different scales. The formula is:

(35) $$\mathrm{MARE}=\frac{1}{n}\sum_{i=1}^{n}\left|\frac{y_{i}-{\hat{y}}_{i}}{y_{i}}\right|$$

Lower MARE values indicate higher predictive accuracy and reliability of the model.

To maximize model performance, we systematically sought a balance between model complexity and prediction accuracy, selecting the optimal hyperparameter configuration (see Table 2). An early stopping strategy was implemented, whereby training automatically halts when the validation error ceases to improve significantly. This ensures that the model concludes training at its optimal point, avoiding performance degradation due to overfitting. Additionally, to mitigate randomness in prediction results, we conducted four independent training runs and averaged their outcomes as the final result.

On the training set, the ILPOBP model achieved the following error metrics: MAE of 1.707, MSE of 5.543, RMSE of 2.356, and MARE of 3.573%. On the test set, the ILPOBP model demonstrated high predictive accuracy, with an MAE of 2.008, MSE of 6.631, RMSE of 2.575, and MARE of 4.106%. Table 3 presents the prediction results for 20 randomly selected samples from the test set, showing relative prediction errors below 4.48%. These results highlight the ILPOBP model’s strong predictive capability for this task.

### 4.2. Performance Comparison Between ILPOBP and Other Models

To comprehensively evaluate the superior performance of the ILPOBP model in predicting the “comprehensive aesthetic value” of automobile front faces, we compared ILPOBP with LPOBP and the traditional BP model on the same dataset. All three models were configured with their optimal parameter settings, as shown in Table 2.

**Figure 7:**
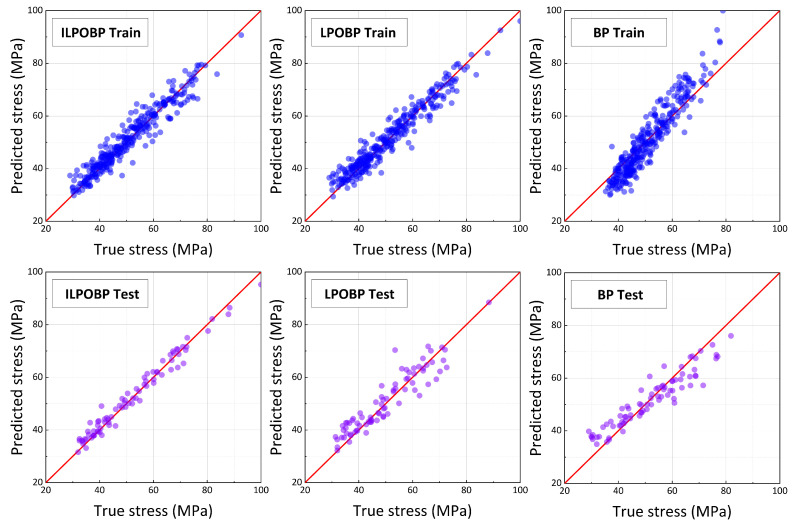
Performance of ILPOBP, LPOBP, and BP models on training and test sets.

As illustrated in Figure 7, the performance of the three models on both the training and test sets shows significant differences. The ILPOBP model outperforms the other two models on both the training and test sets. Particularly on the test set, it achieves a remarkably low relative error (MARE) of 4.106%, with excellent fitting performance in the regression plots. In contrast, while the LPOBP model performs well on the training set due to optimization by the LPO algorithm, its test set errors increase substantially, indicating limited generalization capability. The traditional BP model performs the worst, with higher errors on both the training and test sets compared to ILPOBP and LPOBP, further highlighting its limitations in predictive tasks.

Specifically, Table 4 provides the error metrics for the three models on the same dataset. For the ILPOBP model, the mean absolute error (MAE) on the training set is 1.707, the mean squared error (MSE) is 5.543, the root mean squared error (RMSE) is 2.356, and the mean absolute relative error (MARE) is 3.573%. On the test set, the ILPOBP model achieves an MAE of 2.008, MSE of 6.631, RMSE of 2.575, and MARE of 4.106%. These results demonstrate that ILPOBP not only exhibits high accuracy and stability on the training set but also achieves significantly improved generalization capability on the test set.

The error metrics for the LPOBP model on the training set were MAE 2.284, MSE 10.437, RMSE 2.422, and MARE 3.796%. On the test set, the metrics were MAE 3.478, MSE 20.873, RMSE 4.569, and MARE 7.329%. While LPOBP’s performance on the training set was close to that of ILPOBP, its errors increased significantly on the test set, indicating weaker predictive capability when dealing with new data.

For the BP model, the training set errors were MAE 3.534, MSE 25.635, RMSE 4.458, and MARE 7.942%. On the test set, the errors were even higher, with MAE 4.126, MSE 29.889, RMSE 5.457, and MARE 9.37%. The BP model’s errors were markedly higher than those of both ILPOBP and LPOBP, highlighting its limitations in learning complex data features and its inability to effectively capture the aesthetic characteristics of automobile front faces.

The superior performance of the ILPOBP model can be attributed to the effective incorporation of the MLHD strategy. The MLHD method’s uniform coverage of the sample space significantly enhanced the BP neural network’s ability to learn the data distribution. This allowed the model to more accurately capture the subtle aesthetic features of automobile front faces within a complex, high-dimensional feature space, enabling more precise predictions of the “comprehensive aesthetic value” of automobile front faces. Furthermore, the optimization strategy of the ILPOBP model accelerated model convergence, improved training efficiency, and effectively suppressed overfitting.

As a result, compared to the LPOBP and BP models, the ILPOBP model achieved more accurate and stable predictions of the “comprehensive aesthetic value” on the test set. This comparison underscores the critical role of optimization strategies in enhancing the generalization ability of neural network models and validates the potential of MLHD in complex predictive tasks.

### 4.3. Explainability Analysis of the ILPOBP Model

In product aesthetic prediction, model explainability not only enhances the credibility of prediction results but also provides a key perspective for understanding the model’s working mechanism and revealing the inherent relationship between design features and aesthetic evaluation. This study employs the game theory-based SHAP method [43] to conduct a systematic explainability analysis of the BP neural network model optimized by the improved LPO algorithm (ILPOBP), aiming to quantify the contribution of each aesthetic indicator to the model’s prediction results.

During the SHAP explainability analysis, we used the Shapley value from game theory to measure the impact of each aesthetic feature on the model’s prediction outcome. The formula for calculating the Shapley value is as follows:

(36) $$\phi_{i}=\sum_{S\subseteq N\setminus \{i\}}\frac{|S|!(|N|-|S|-1)!}{|N|!}\left[f(S\cup \{i\})-f(S)\right]$$

where $\phi_{i}$ represents the Shapley value of feature *i*, reflecting the importance of that feature to the prediction result; *S* is a subset of features, *N* is the set of all features, f(S) denotes the model’s prediction when only considering the feature subset *S*, and |S| represents the size of the feature subset.

By calculating the Shapley values, we systematically analyzed the influence of aesthetic indicators on the prediction of the “comprehensive aesthetic value” of car front faces within the ILPOBP model. The results indicate that key indicators such as symmetry, regularity, and similarity significantly contribute to the model’s predictions. These features act as primary predictors, collectively enhancing the accuracy of the predictions while ensuring the stability and rationality of the ILPOBP model.

**Figure 8:**
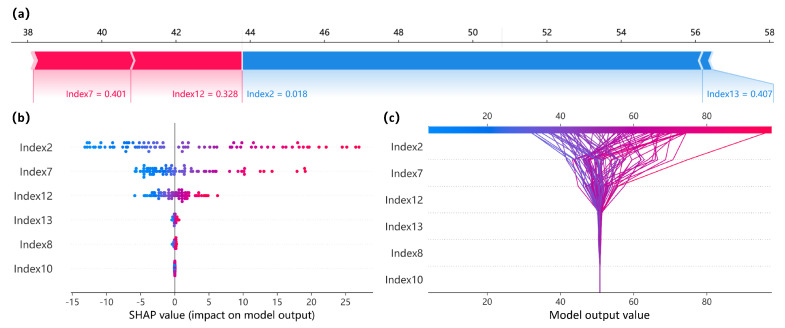
Quantitative analysis of the overall aesthetic value of car front faces based on SHAP. (**a**) SHAP force plot, showing the contribution of each aesthetic indicator to the prediction of an individual sample; (**b**) SHAP summary plot, showing the contribution distribution and importance ranking of aesthetic indicators on the test set; (**c**) SHAP decision plot, showing the cumulative effect of aesthetic indicators on the model output.

Figure 8 presents the quantitative SHAP value analysis of aesthetic indicators concerning the “comprehensive aesthetic value” of car front faces. The SHAP value distribution plot and feature impact trajectory diagram visually demonstrate the positive and negative effects of different indicators and their impact paths on the model’s output. This facilitates the understanding of interactions between features and their performance under various conditions. Specifically, symmetry plays a crucial role in aesthetic evaluation. SHAP value analysis within the model reveals that symmetry (Index2) exerts both significant positive and negative influences on the comprehensive aesthetic value, establishing it as a key predictor and validating the importance of symmetry emphasized in design theory. Moreover, regularity (Index7) also makes a significant positive contribution to the predictions, reflecting the importance of orderliness and structure in visual aesthetics. The contribution of similarity (Index12) underscores that similar shapes and structures in car front face design evoke a sense of familiarity and harmony, thereby enhancing overall aesthetic evaluations. In contrast, the contributions of common directionality (parallelism) (Index8) and attractiveness (Index10) are relatively minor, indicating their limited role in predictions. Through this analysis, we confirmed the critical importance of these indicators as dominant features in model predictions.

### 4.4. Ablation Study on High-Contribution Aesthetic Indicators

To further verify that the ILPOBP model captures structural regularities consistent with principles of formal aesthetics, rather than incidental statistical patterns in the dataset, we conduct an ablation study on the high-contribution indicators identified in Section 2.3 and Section 4.4. In particular, we focus on three indicators: degree of symmetry (D2), degree of regularity (D7), and degree of similarity (D12), which exhibit high contributions in both the correlation analysis (Table 1) and the SHAP-based importance analysis (Figure 8).

In terms of experimental setup, we start from the complete feature subset consisting of all six sensitive indicators (D2, D7, D8, D10, D12, D13), and progressively remove the high-contribution indicators according to the following steps: (1) Remove similarity (D12); (2) Based on (1), further remove regularity (D7); (3) Based on (2), further remove symmetry (D2). For each feature configuration, the ILPOBP model is retrained under the same data split, network architecture, and hyperparameter settings, and evaluated on the test set. Each configuration is independently run four times, and the mean values are reported as the final performance metrics. To quantify performance degradation, we report the test-set MARE and RMSE, as well as their changes relative to the full-feature model:

(37) $$\Delta {\mathrm{MARE}}_{k}={\mathrm{MARE}}_{k}-{\mathrm{MARE}}_{\mathrm{full}}$$

(38) $$\Delta {\mathrm{RMSE}}_{k}={\mathrm{RMSE}}_{k}-{\mathrm{RMSE}}_{\mathrm{full}}$$

where “full” denotes the ILPOBP model using all six sensitive indicators, and “k” denotes a specific ablation configuration.

As shown in Table 5, when only the similarity indicator D12 is removed, the model performance has already degraded to some extent: MARE increases by approximately 2.213 percentage points, and RMSE increases by about 0.444. When the regularity indicator D7 is further removed on this basis, the performance degradation becomes more pronounced (ΔMARE = 6.870, ΔRMSE = 4.132), indicating that regularity provides complementary discriminative information to similarity in characterizing structural orderliness. When symmetry D2 is additionally removed, the performance drop is the most substantial, with ΔMARE exceeding 17.886 percentage points and ΔRMSE increasing by more than 11.144. This suggests that symmetry plays a central structural-constraining role in the constructed morphological feature space. This phenomenon is highly consistent with the emphasis placed by formal aesthetics and Gestalt perceptual theory on visual organizational principles such as “symmetry,” “regularity,” and “similarity.”

## 5. Discussion

While the proposed ILPOBP model demonstrates high predictive accuracy and interpretability, the dataset size of approximately 400 automobile front face images warrants discussion. In the era of data-intensive deep learning, such a sample size might initially appear limited, especially given the inherent subjectivity of aesthetic evaluation. However, unlike end-to-end convolutional neural networks that require massive datasets to learn from high-dimensional raw pixels, our approach relies on rigorous expert-driven feature engineering. By mathematically reducing structural aesthetics to only six highly sensitive morphological indicators (e.g., Symmetry, Regularity), the input space becomes extremely low-dimensional. For our optimized BP neural network with only six input nodes, a curated dataset of 400 instances provides sufficient data density to effectively map non-linear aesthetic relationships without the risk of severe overfitting. Furthermore, to mitigate subjective bias, the labels for these samples are derived from a rigorous game-theoretic fusion of aggregated expert consensus and objective geometric variance (CVM-TOPSIS), ensuring a high signal-to-noise ratio. Nevertheless, acknowledging cross-domain aesthetic variability, future research will aim to construct larger, multi-modal databases across diverse product categories to further validate and expand this intelligent evaluation framework.

Beyond dataset considerations, translating the model’s interpretability into actionable design guidance represents a vital frontier for intelligent product design. While this study successfully utilizes SHAP to reveal global aesthetic mapping rules (e.g., the overarching correlation of symmetry and regularity with higher aesthetic scores), industrial design practice ultimately demands instance-level diagnostic feedback for specific conceptual drafts. Translating these global insights into precise design interventions—such as employing local SHAP visualizations to explicitly pinpoint that a low regularity score in a specific car draft necessitates realigning the lower grille elements—is the key to replacing intuition-based trial-and-error with data-driven optimization. Building upon the predictive and interpretable foundation established in this study, developing a comprehensive, automated evaluate-diagnose-optimize closed-loop pipeline serves as a highly promising direction for our subsequent research. Ultimately, integrating this interpretable diagnostic framework directly into intelligent computer-aided design (CAD) platforms will fully unlock its practical value in generative design workflows.

Furthermore, since aesthetic evaluation is inherently a visual task, it is essential to contextualize our expert-driven approach alongside modern end-to-end deep learning architectures. While Convolutional Neural Networks (CNNs) and Vision Transformers (ViTs) excel at capturing extremely rich, implicit visual patterns directly from raw images, as demonstrated in recent aesthetic and material design studies [44,45], a fundamental trade-off exists between representational power and design interpretability. The black-box nature of pure deep models makes it highly challenging to translate high-dimensional latent vectors into explicit, actionable modification guidelines. In contrast, our reliance on hand-crafted features grounded in Gestalt psychology prioritizes structural interpretability; it guarantees that every input dimension possesses a clear semantic meaning, thereby enabling the transparent diagnostic feedback demonstrated by the SHAP analysis. Recognizing that purely manual features may limit scalability in unconstrained tasks, we envision that the most promising future trajectory lies in hybrid architectures. Fusing explicit, interpretable geometric priors with the rich representations extracted by modern vision models will harness their extraordinary data-driven perceptual power without sacrificing the structural transparency essential for actionable intelligent design.

## 6. Conclusions

This study proposes a machine learning method based on sensitive morphological aesthetic features for the evaluation and prediction of product form aesthetics. The specific contributions are as follows:

1. Based on formal aesthetic principles and Gestalt laws, this study constructs a comprehensive aesthetic indicator system and corresponding computational formulas, forming a framework for visual aesthetic evaluation. Subjective and objective weights were determined using the grey relational analysis method and the coefficient of variation-TOPSIS method, respectively, and integrated through a game-theory-based combination weighting approach to build a comprehensive aesthetic evaluation model. Sensitivity analysis using Pearson correlation coefficients identified six key aesthetic indicators for model training, reducing the complexity of the evaluation model and enhancing analytical efficiency.
2. Utilizing the above dataset, an improved ILPOBP model optimized with the MLHD strategy was proposed. This model demonstrates superior predictive accuracy and generalization ability when handling complex, high-dimensional data. Experimental results indicate that the ILPOBP model significantly outperforms comparative models in major error metrics (e.g., MAE, MSE, RMSE, and MARE) on both the training and testing datasets, thereby validating its excellence in aesthetic evaluation tasks. These results confirm that the introduction of the MLHD strategy effectively enhances the model’s stability and predictive performance.
3. To enhance the model’s interpretability, a systematic analysis was conducted using the SHAP method to uncover the contributions of key features to aesthetic predictions. The findings highlight that features such as symmetry, regularity, and similarity have significant positive impacts within the model. This interpretability analysis not only improves model transparency and deepens understanding of its decision-making mechanisms but also provides designers with clear and actionable guidance for product design optimization.

## Figures and Tables

**Table 1:** Correlation coefficients of 17 aesthetic indicators.

Aesthetic Indicators	Correlation Coefficient	Aesthetic Indicators	Correlation Coefficient
Symmetry Degree (D2)	0.534480234	Continuity Degree (D9)	0.136226141
Regularity Degree (D7)	0.381353647	Proportionality Degree (D3)	0.125604031
Common Direction Degree (D8)	0.264295782	Hierarchy Degree (D15)	0.036547506
Similarity Degree (D12)	0.2533643	Simplification Degree (D11)	−0.004833881
Attractiveness Degree (D10)	0.236165742	Contrast Degree (D16)	−0.014365683
Proportional Similarity Degree (D13)	0.218901525	Balance Degree (D1)	−0.045705435
Wholeness Degree (D6)	0.20453097	Rhythm Degree (D4)	−0.201463031
Stability Degree (D14)	0.164520415	Complexity Degree (D17)	−0.254947003
Orderliness Degree (D5)	0.136831441	–	–

**Table 2:** Hyperparameter configurations for three artificial neural networks.

	ILPOBP	LPOBP	BP
Input	6	6	6
Output	1	1	1
Number of hidden layers	1	1	1
Number of neurons per layer	13	13	13
Learning rate	0.001	0.001	0.001
Number of search agents	30	30	/
Maximum Iterations	50	50	/
Activation function	Sigmoid	Sigmoid	Sigmoid

**Table 3:** Prediction results of 20 random samples from the test set using the ILPOBP model.

Sample	True Score	Predicted Score	Relative Error
Sample 1	53.4921	52.0933	2.6148
Sample 2	37.6708	37.8239	0.4062
Sample 3	69.0698	70.5931	2.2054
Sample 4	68.7477	70.7749	2.9488
Sample 5	43.4009	42.8919	1.1726
Sample 6	72.0413	70.3416	2.3594
Sample 7	69.4395	68.7581	0.9814
Sample 8	54.6957	54.7323	0.0669
Sample 9	88.3692	86.4536	2.1678
Sample 10	57.4335	56.6908	1.2932
Sample 11	40.3965	40.6137	0.5376
Sample 12	63.4909	66.3104	4.4409
Sample 13	63.1417	60.9226	3.5145
Sample 14	50.8816	50.1763	1.3861
Sample 15	49.4225	51.2899	3.7783
Sample 16	60.1299	59.2227	1.5089
Sample 17	48.3261	48.4761	0.3104
Sample 18	72.3347	71.5977	1.0189
Sample 19	40.0685	39.1119	2.3874
Sample 20	87.9038	83.9609	4.4853
Sample 21	68.4273	70.3793	2.8526
Sample 22	52.9368	53.4689	1.0051
Sample 23	74.2605	76.4674	2.9718
Sample 24	89.5142	90.3383	0.9206
Sample 25	40.7589	39.3569	3.4397
Sample 26	81.4067	80.2646	1.4029
Sample 27	66.3284	68.3467	3.0428
Sample 28	48.1927	49.8963	3.5349
Sample 29	72.7352	73.3578	0.8559
Sample 30	59.0463	60.5785	2.5949
Sample 31	85.1625	84.8964	0.3124
Sample 32	62.4087	60.9866	2.2786
Sample 33	77.9530	75.3234	3.3733
Sample 34	44.2874	43.6545	1.4291
Sample 35	54.0196	53.3423	1.2538
Sample 36	69.8013	69.0124	1.1302
Sample 37	43.3627	43.7536	0.9014
Sample 38	87.2459	86.2134	1.1834
Sample 39	46.7104	47.3445	1.3575
Sample 40	79.6398	80.2344	0.7466

**Table 4:** Performance of ILPOBP and two other models on the same dataset.

Model	Data Set	MAE	MSE	RMSE	MARE (%)
ILPOBP	Train set	1.707	5.543	2.356	3.573
	Test set	2.008	6.631	2.575	4.106
LPOBP	Train set	2.284	10.437	2.422	3.796
	Test set	3.478	20.873	4.569	7.329
BP	Train set	3.534	25.635	4.458	7.942
	Test set	4.126	29.889	5.457	9.370

**Table 5:** Ablation results of high-contribution aesthetic indicators for the ILPOBP model on the test set.

Feature Configuration	MARE (%)	RMSE	ΔMARE (%)	ΔRMSE
D2, D7, D8, D10, D12, D13	4.106	2.575	0.000	0.000
– D12	6.319	3.019	2.213	0.444
– D12, D7	10.976	6.707	6.870	4.132
– D12, D7, D2	21.992	13.719	17.886	11.144

## Data Availability

The data presented in this study are available on request from the corresponding author.

## Appendix A. Calculation Steps for the 17 Aesthetic Metrics

### Appendix A.1. Balance Degree

Evaluates the equilibrium of visual factors by quantifying the difference in visual gravitational moments of morphological elements on either side of the contour’s center.

(A1) $$D_{1}=1-\frac{\left|D_{1y}\right|+\left|D_{1x}\right|}{2}$$

(A2) $$D_{1y}=\frac{w^{L}-w^{R}}{\max\left(\left|w^{L}\right|,\left|w^{R}\right|\right)}$$

(A3) $$D_{1x}=\frac{w^{T}-w^{B}}{\max(|w^{T}|,|w^{B}|)}$$

(A4) $$w^{k}=\sum_{i=1}^{h}\sum_{j=1}^{b}d_{ij}^{k}$$

In the formula: $D_{1}$ represents the balance degree; $D_{1y}$ and $D_{1x}$ indicate the vertical and horizontal balance degrees, respectively; $w^{L}$ and $w^{R}$ are the visual gravitational moments on the left and right sides of the vertical symmetry axis, respectively; $w^{T}$ and $w^{B}$ are the visual gravitational moments on the upper and lower sides of the horizontal symmetry axis, respectively. The parameter *k* stands for L,R,T,B, corresponding to the left, right, top, and bottom regions divided by the symmetry axes. Additionally, $d_{ij}$ represents the distance from pixel $P_{ij}$ to the symmetry axis.

### Appendix A.2. Symmetry Degree

Measures the degree of overlap of elements along vertical, horizontal, or rotational axes, with the minimization of differences on either side of the axis or the center point as the standard.

(A5) $$D_{2}=1-\frac{\left|D_{2y}\right|+\left|D_{2x}\right|+\left|D_{2r}\right|}{3}$$

(A6) $$D_{2y}=\frac{\overset{h}{\underset{i=1}{\Sigma }}\overset{b/2}{\underset{j=1}{\Sigma }}\left|d_{ij}^{L}-d_{ij}^{R}\right|}{\max\left(\left|\overset{h}{\underset{i=1}{\Sigma }}\overset{b/2}{\underset{j=1}{\Sigma }}\left(d_{ij}^{L}\right)\right|,\left|\overset{h}{\underset{i=1}{\Sigma }}\overset{b/2}{\underset{j=1}{\Sigma }}\left(d_{ij}^{R}\right)\right|\right)}$$

(A7) $$D_{2x}=\frac{\overset{b}{\underset{j=1}{\Sigma }}\overset{h/2}{\underset{i=1}{\Sigma }}\left|d_{ji}^{T}-d_{ji}^{B}\right|}{\max\left(\left|\overset{b}{\underset{j=1}{\Sigma }}\overset{h/2}{\underset{i=1}{\Sigma }}\left(d_{ji}^{T}\right)\right|,\left|\overset{b}{\underset{j=1}{\Sigma }}\overset{h/2}{\underset{i=1}{\Sigma }}\left(d_{ji}^{B}\right)\right|\right)}$$

(A8) $$D_{2r}=\frac{\overset{h}{\underset{i=1}{\Sigma }}\overset{b/2}{\underset{j=1}{\Sigma }}\left|d_{ij}^{L}-d_{(h-i+1)j}^{R}\right|}{\max\left(\left|\overset{h}{\underset{i=1}{\Sigma }}\overset{b/2}{\underset{j=1}{\Sigma }}\left(d_{ij}^{L}\right)\right|,\left|\overset{h}{\underset{i=1}{\Sigma }}\overset{b/2}{\underset{j=1}{\Sigma }}\left(d_{ij}^{R}\right)\right|\right)}$$

In the formula: $D_{2}$ represents the symmetry degree; $D_{2y}$, $D_{2x}$, and $D_{2r}$ represent the vertical, horizontal, and rotational symmetry degrees, respectively.

### Appendix A.3. Proportionality Degree

Assesses the deviation in the proportional relationships between morphological elements from classical ratios (1:1, 1:1.414, 1:1.618, 1:1.732, 1:2).

(A9) $$D_{3}=\frac{1}{n}\sum_{i=1}^{n}\left(1-\frac{\min\left(\left|p_{j}-p_{i}\right|\right)}{0.5}\right)$$

(A10) $$p_{i}=\min\left(\frac{h_{i}}{b_{i}},\frac{b_{i}}{h_{i}}\right)$$

In the formula: $D_{3}$ represents the proportion degree; $b_{i}$ and $h_{i}$ denote the width and height of the minimum bounding rectangle of line *i*, respectively; $p_{i}$ represents the minimum value of the ratio of width to height of the minimum bounding rectangle; $p_{i}$ also represents the classical proportion.

### Appendix A.4. Rhythm Degree

Describes the dynamism and regularity generated when morphological elements repeat in a pattern through their arrangement, size ratios, quantity distribution, and form variations.

(A11) $$D_{4}=1-\frac{|D_{4x}\left|+\right|D_{4y}|}{2}$$

(A12) $$D_{4x}=\left(\begin{array}{c} |X_{UL}^{\prime }-X_{UR}^{\prime }\left|+\right|X_{UL}^{\prime }-X_{LR}^{\prime }|+\\ |X_{UL}^{\prime }-X_{LL}^{\prime }\left|+\right|X_{UR}^{\prime }-X_{LR}^{\prime }|+\\ |X_{UR}^{\prime }-X_{LL}^{\prime }\left|+\right|X_{LR}^{\prime }-X_{LL}^{\prime }| \end{array}\right)/6$$

(A13) $$X_{j}=\sum_{i=1}^{n_{j}}x_{ij}$$

(A14) $$X_{j}^{\prime }=\frac{X_{j}-X_{\min}}{X_{\max}-X_{\min}}$$

(A15) $$D_{4y}=\left(\begin{array}{c} |Y_{UL}^{\prime }-Y_{UR}^{\prime }\left|+\right|Y_{UL}^{\prime }-Y_{LR}^{\prime }|+\\ |Y_{UL}^{\prime }-Y_{LL}^{\prime }\left|+\right|Y_{UR}^{\prime }-Y_{LR}^{\prime }|+\\ |Y_{UR}^{\prime }-Y_{LL}^{\prime }\left|+\right|Y_{LR}^{\prime }-Y_{LL}^{\prime }| \end{array}\right)/6$$

(A16) $$Y_{j}=\sum_{i=1}^{n_{j}}y_{ij}$$

(A17) $$Y_{j}^{\prime }=\frac{Y_{j}-Y_{\min}}{Y_{\max}-Y_{\min}}$$

In the formula: $D_{4}$ represents the rhythm degree; $D_{4x}$ and $D_{4y}$ denote the degree of variation in the positions of elements in each region along the *x*- and *y*-directions, respectively; $x_{ij}$ indicates the *x*-coordinate (i.e., the distance from the *y*-axis) of pixel *i* in quadrant *j*; *j* represents the four quadrants: UL (upper left), UR (upper right), LL (lower left), and LR (lower right).

### Appendix A.5. Orderliness Degree

Reflects the extent to which the positional layout of product morphological elements aligns with the viewer’s eye movement path.

(A18) $$D_{5}=1-\frac{\sum |q_{j}-v_{j}|}{8}$$

(A19) $$v_{j}=\left\{\begin{array}{cc} 4, & \mathrm{if}\;w_{j}=\max\;\mathrm{in}\;w\\ 3, & \mathrm{if}\;w_{j}=2\mathrm{nd}\;\mathrm{in}\;w\\ 2, & \mathrm{if}\;w_{j}=3\mathrm{rd}\;\mathrm{in}\;w\\ 1, & \mathrm{if}\;w_{j}=\min\;\mathrm{in}\;w \end{array}\right.$$

(A20) $$w_{j}=q_{j}\sum_{i=1}^{h_{j}}\sum_{j=1}^{b_{j}}p_{ij}^{N}$$

In the formula: $D_{5}$ represents the order degree; $p_{ij}^{N}$ denotes the number of pixels at point $P_{ij}$; $q_{j}$ represents the weights of the four quadrants—UL, UR, LL, and LR—which are 4, 3, 2, and 1, respectively.

### Appendix A.6. Wholeness Degree

Measures the compactness of the layout of elements.

(A21) $$D_{6}=1-\frac{a_{g}-\sum_{i=1}^{n}a_{i}}{a_{0}-\sum_{i=1}^{n}a_{i}}$$

In the formula: $D_{6}$ represents the degree of wholeness; $a_{i}$ denotes the area of the convex hull of a line; $a_{g}$ is the area of the minimum bounding rectangle of a group of elements; and $a_{0}$ represents the area of the contour shape.

### Appendix A.7. Regularity Degree

Evaluates the alignment of elements, reflecting the tidiness and regularity of the morphology.

(A22) $$D_{7}=\frac{|D_{7s}\left|+\right|D_{7c}|}{2}$$

(A23) $$D_{7s}=1-\frac{n_{vs}+n_{hs}}{4n}$$

(A24) $$D_{7c}=1-\frac{n_{vc}+n_{hc}}{2n}$$

In the formula: $D_{7}$ represents the degree of regularity; $D_{7s}$ denotes the alignment degree of elements in the left, right, top, and bottom directions; $D_{7c}$ indicates the alignment degree of the centroids of elements in the horizontal and vertical directions; $n_{vs}$ and $n_{hs}$ represent the number of tangents for all elements in the vertical and horizontal directions, respectively (overlapping lines are counted as a single line). Let *n* denote the number of lines. When none of the lines are aligned, the total number of tangents in the vertical and horizontal directions (left, right, top, and bottom) is 4n. When all lines are fully aligned, this number reduces to 4, meaning that the minimum bounding rectangles of the *n* elements completely overlap.

### Appendix A.8. Common Direction Degree (Parallelism)

Assesses the grouping extent of morphological elements within the contour that exhibit a shared directionality.

Let $n_{g}$ represent the number of key points. By connecting any two key points, the total number of connections is:

(A25) $$\left(n_{g}-1\right)+\left(n_{g}-2\right)+\left(n_{g}-3\right)+\cdots +2+1=n_{g}\times \left(n_{g}-1\right)/2$$

(A26) $$D_{8}=\frac{m_{ts}-m_{tg}}{\frac{n_{g}\times \left(n_{g}-1\right)}{2}}=\frac{2\left(m_{ts}-m_{tg}\right)}{n_{g}\times \left(n_{g}-1\right)}$$

In the formula: $D_{8}$ represents the degree of common direction; $m_{ts}$ denotes the number of pairs of parallel connections; and $m_{tg}$ is the number of groups with a common directional alignment.

### Appendix A.9. Continuity Degree

Measures the grouping extent of morphological elements within the contour that demonstrate visual continuity.

(A27) $$D_{9}=\frac{m_{1s}}{n_{g}}$$

In the formula: $D_{9}$ represents the degree of continuity; $m_{1s}$ denotes the number of continuous lines (maximized when all key points are connected along a single line, with $m_{1s}$ reaching a maximum of $n_{g}$).

### Appendix A.10. Attractiveness Degree

Evaluates the strength of the visual attraction exerted by key points in the contour on observers.

(A28) $$D_{10}=1-\frac{1}{n_{g}}$$

In the formula: $D_{10}$ represents the degree of attractiveness, and $n_{g}$ denotes the number of key points.

### Appendix A.11. Simplification Degree

Measures the degree of visual simplification grouping of morphological elements within the contour.

(A29) $$D_{11}=\frac{n_{si}-n_{sg}}{n}$$

In the formula: $D_{11}$ represents the degree of simplification; $n_{si}$ denotes the number of elements in the simplified groups, and $n_{sg}$ represents the number of simplified groups.

### Appendix A.12. Similarity Degree

Assesses the grouping extent of morphological elements within the contour that exhibit visual similarity

(A30) $$D_{12}=\frac{1}{n(n-1)}\sum_{i=1}^{n}\sum_{j=1}^{n}D_{ij},\;i\neq j$$

(A31) $$D_{ij}=\frac{\cos\theta_{ij}}{|R_{ij}-1|+1}$$

(A32) $$\cos\theta_{ij}=\frac{\overset{m}{\underset{k=1}{\Sigma }}p_{ik}\cdot p_{jk}}{\sqrt{\overset{m}{\underset{k=1}{\Sigma }}p_{ik}^{2}}\cdot \sqrt{\overset{m}{\underset{k=1}{\Sigma }}p_{jk}^{2}}}$$

(A33) $$R_{ij}=\frac{\left|p_{ik}\right|}{\left|p_{jk}\right|}=\frac{\sqrt{\overset{m}{\underset{k=1}{\Sigma }}p_{ik}^{2}}}{\sqrt{\overset{m}{\underset{k=1}{\Sigma }}p_{jk}^{2}}}$$

In the formula: $D_{12}$ represents the degree of similarity, and $\cos\theta_{ij}$ denotes the cosine similarity.

### Appendix A.13. Proportional Similarity Degree

Evaluates the degree of similarity in the width-to-height ratio of elements.

(A34) $$D_{13}=\frac{1}{n(n-1)/2}\times \sum_{i=1}^{n}\sum_{\begin{array}{c} j=1\\ i\neq j \end{array}}^{n}t_{ij}=\frac{2}{n(n-1)}\sum_{i=1}^{n}\sum_{j=1}^{n}t_{ij},\;i\neq j$$

(A35) $$t_{ij}=\min\left(\frac{h_{i}/b_{i}}{h_{j}/b_{j}},\frac{h_{j}/b_{j}}{h_{i}/b_{i}}\right)$$

In the formula: $D_{13}$ represents the degree of similarity in proportions; $t_{ij}$ denotes the minimum width-to-height ratio of the bounding rectangles for lines *i* and *j*; $b_{i}$, $h_{i}$, $b_{j}$, and $h_{j}$ represent the width and height of the minimum bounding rectangles of contours *i* and *j*, respectively.

### Appendix A.14. Stability Degree

Assesses the visual stability of morphological elements within the contour based on the height of the center of gravity, its deviation from the symmetry axis, and the size of the supporting area.

(A36) $$D_{14}=\frac{D_{14y}+D_{14x}+D_{14b}}{3}$$

(A37) $$D_{14y}=\frac{1}{2}-\frac{y_{c}}{h_{c}}$$

(A38) $$D_{14x}=1-\frac{2|x_{c}|}{b_{c}}$$

(A39) $$D_{14b}=\frac{x_{b}}{b_{c}}$$

In the formula: $D_{14}$ represents the degree of stability; $D_{14y}$, $D_{14x}$, and $D_{14b}$ denote the effects of center of gravity height, lateral offset, and support surface size on stability, respectively. $b_{c}$ and $h_{c}$ represent the width and height of the product contour, respectively; $x_{b}$ indicates the width of the support surface; and $x_{c}$ and $y_{c}$ denote the *x*- and *y*-coordinates of the product’s center of gravity, respectively.

### Appendix A.15. Hierarchy Degree

Measures how the size, distance, and relative position of design elements clarify their hierarchical relationships and enhance visual diversity.

(A40) $$D_{15}=1-\frac{1}{2m}-\frac{1}{2}\sum_{i=1}^{m}\frac{S_{i}^{F}-S_{i}^{L}}{S_{1}}$$

In the formula: $D_{15}$ represents the degree of hierarchy; $S_{i}^{F}$ and $S_{i}^{L}$ denote the lengths of the first and last lines at the *i*-th hierarchical level, respectively.

### Appendix A.16. Contrast Degree

Reflects the degree of variation in form and size among design elements.

(A41) $$D_{16}=1-\frac{2}{m\;(m-1)}\sum_{i=1}^{m}\frac{\frac{1}{k}\sum_{k}S_{i+1}^{k}}{\frac{1}{j}\sum_{j}S_{i}^{j}}$$

In the formula: $D_{16}$ represents the degree of contrast; $S_{i}^{j}$ denotes the length of the *j*-th line at the *i*-th hierarchical level; $S_{i+1}^{k}$ denotes the length of the *k*-th line at the i+1-th hierarchical level; $\frac{1}{j}\sum_{j}S_{i}^{j}$ represents the average length of lines at the *i*-th level; and $\frac{1}{k}\sum_{k}S_{i+1}^{k}$ represents the average length of lines at the i+1-th level.

### Appendix A.17. Complexity Degree

Measures the curvature of the contour by describing the relative increment in perimeter compared to its convex hull perimeter.

(A42) $$D_{17}=\frac{1}{n}\sum_{i=1}^{n}\left(1-\frac{S_{i}^{T}}{S_{i}^{L}}\right)$$

In the formula: $D_{17}$ represents the degree of complexity; $S_{i}^{L}$ and $S_{i}^{T}$ denote the contour perimeter and the convex hull perimeter of the *i*-th curve, respectively.

## References

[1] Noble C.H., Kumar M. (2010). Exploring the appeal of product design: A grounded, value-based model of key design elements and relationships. J. Prod. Innov. Manag..

[2] Nawar S.H., Etawy M.S., Nassar G.E., Mohammed N., Hassabo A.G. (2024). The impact of cmf design on product design. J. Text. Color. Polym. Sci..

[3] Wang Y., Song F., Liu Y., Li Y., Wang W. (2025). Research on the association mechanism and evaluation model between EMG data and product aesthetic quality in product aesthetics evaluation. J. Eng. Des..

[4] Cheng P., Mugge R., de Bont C. (2018). Transparency in product design: Investigating design intentions and consumers’ interpretations. J. Eng. Des..

[5] Wang Z., Duff B.R.L., Clayton R.B. (2018). Establishing a factor model for aesthetic preference for visual complexity of brand logo. J. Curr. Issues Res. Advert..

[6] Zen M., Vanderdonckt J. Assessing user interface aesthetics based on the inter-subjectivity of judgment. Proceedings of the 30th International BCS Human Computer Interaction Conference.

[7] Liu S., Xiang Z., Yao H., Cong J. (2024). A novel data-driven method for product aesthetics evaluating and optimising based on knowledge graph. J. Eng. Des..

[8] Jin S. (2023). Research on improvement of comprehensive evaluation method of appearance elements design of elderly products. Int. J. Interact. Des. Manuf..

[9] Miyahara K. (2014). Exploring social aesthetics: Aesthetic appreciation as a method for qualitative sociology and social research. Int. J. Jpn. Soc..

[10] Bo Y., Yu J., Zhang K. (2018). Computational aesthetics and applications. Vis. Comput. Ind. Biomed. Art.

[11] Luo S., Shan P., Bian Z., Lin H., Zhang Y., Cui Z., Shen C., Wang L. (2024). Effects of product personalisation degree on user perception in car front design. J. Eng. Des..

[12] Schindler I., Hosoya G., Menninghaus W., Beermann U., Wagner V., Eid M., Scherer K.R. (2017). Measuring aesthetic emotions: A review of the literature and a new assessment tool. PLoS ONE.

[13] South L., Saffo D., Vitek O., Dunne C., Borkin M.A. (2022). Effective use of Likert scales in visualization evaluations: A systematic review. Comput. Graph. Forum.

[14] Ma C., Chen C., Liu Q., Gao H., Li Q., Gao H., Shen Y. (2017). Sound quality evaluation of the interior noise of pure electric vehicle based on neural network model. IEEE Trans. Ind. Electron..

[15] Steenis N.D., Van Herpen E., Van Der Lans I.A., Ligthart T.N., Van T., Hans C.M. (2017). Consumer response to packaging design: The role of packaging materials and graphics in sustainability perceptions and product evaluations. J. Clean. Prod..

[16] Noorjahan S., Basha S.S. (2025). The Laplacian energy of an intuitionistic fuzzy rough graph and its utilisation in decision-making. Oper. Res. Decis..

[17] Noorjahan S., Basha S.S. (2024). Developing an intuitionistic fuzzy rough new correlation coefficient approach for enhancing robotic vacuum cleaner. Sci. Prog..

[18] Trumpler R.J., Weaver H.F. (2023). Statistical Astronomy.

[19] Zhou L., Xue C., Tang W., Li J., Niu Y. (2013). Aesthetic evaluation method of interface elements layout design. J. Comput.-Aided Des. Comput. Graph..

[20] Zhou A., Ouyang J., Su J., Zhang S., Yan S. (2020). Multimodal optimisation design of product forms based on aesthetic evaluation. Int. J. Arts Technol..

[21] Wang Z., Pan H., Li J.-S., Niu S.-F. (2024). Exploring product rendering generation design catering to multi-emotional needs through the Superiority Chart-Entropy Weight method and Stable Diffusion model. Adv. Eng. Inform..

[22] Deng L., Wang G. (2020). Quantitative Evaluation of Visual Aesthetics of Human-Machine Interaction Interface Layout. Comput. Intell. Neurosci..

[23] Noorjahan S., Basha S.S. (2024). Decision-making using the correlation coefficient measures of intuitionistic fuzzy rough graph. Hacet. J. Math. Stat..

[24] Shaik N., Shaik S.B. (2025). Wiener index application in intuitionistic fuzzy rough graphs for transport network flow. Sci. Rep..

[25] AlShafeey M., Csáki C. (2021). Evaluating neural network and linear regression photovoltaic power forecasting models based on different input methods. Energy Rep..

[26] Fu L., Lei Y., Zhu L., Lv J. (2024). An evaluation and design method for Ming-style furniture integrating Kansei engineering with particle swarm optimization-support vector regression. Adv. Eng. Inform..

[27] Charbuty B., Abdulazeez A. (2021). Classification based on decision tree algorithm for machine learning. J. Appl. Sci. Technol. Trends.

[28] Sun L., Ji Y., Zhu X., Peng T. (2022). Process knowledge-based random forest regression for model predictive control on a nonlinear production process with multiple working conditions. Adv. Eng. Inform..

[29] Wang Z., Lv Z., Wang J., You F. (2025). IXAI: Generative design of automotive styling based on inception convolution with explainable AI. J. Eng. Des..

[30] Chou J.-R. (2011). A Gestalt–Minimalism-based decision-making model for evaluating product form design. Int. J. Ind. Ergon..

[31] Lugo J., Schmiedeler J.P., Batill S.M., Carlson L. (2016). Relationship between product aesthetic subject preference and quantified gestalt principles in automobile wheel rims. J. Mech. Des..

[32] Ngo D.C.L., Teo L.S., Byrne J.G. (2003). Modelling interface aesthetics. Inf. Sci..

[33] Chen P. (2019). A novel coordinated TOPSIS based on coefficient of variation. Mathematics.

[34] Behzadian M., Otaghsara S.K., Yazdani M., Ignatius J. (2012). A state-of the-art survey of TOPSIS applications. Expert Syst. Appl..

[35] Rao G.S., Aslam M., Alamri F.S., Jun C.-H. (2024). Comparing the efficacy of coefficient of variation control charts using generalized multiple dependent state sampling with various run-rule control charts. Sci. Rep..

[36] Li S., Zheng H., Han T., Hu J., Zhang C., Yu C. (2024). Enhanced Presence Evaluation in Virtual Reality Feedback System with TOPSIS Model. Int. J. Hum.-Interact..

[37] Li Q., Liu Z., Yang Y., Han Y., Wang X. (2023). Evaluation of water resources carrying capacity in Tarim River Basin under game theory combination weights. Ecol. Indic..

[38] Maschler M., Zamir S., Solan E. (2020). Game Theory. https://books.google.fr/books?hl=en&lr=&id=o6UlEAAAQBAJ&oi=fnd&pg=PR14&dq=Maschler,+M.%3B++Zamir,+S.%3B++Solan,+E.+/%5Chl%7BGame+theory.%7D&ots=HlESJpSwhd&sig=evAAJnIEVRzn5ucGQuIkD0OKQZk&redir_esc=y#v=onepage&q&f=false.

[39] Obilor E.I., Amadi E.C. (2018). Test for significance of Pearson’s correlation coefficient. Int. J. Innov. Math. Stat. Energy Policies.

[40] Ghasemi M., Zare M., Zahedi A., Trojovskỳ P., Abualigah L., Trojovská E. (2024). Optimization based on performance of lungs in body: Lungs performance-based optimization (LPO). Comput. Methods Appl. Mech. Eng..

[41] Gao Z., Zhu C., Shu Y., Wang S., Wang C., Chen Y. (2024). Predicting the low-cycle fatigue life of Ti-6Al-4V alloy using backpropagation neural network optimized by the improved dung beetle algorithm. Fatigue Fract. Eng. Mater. Struct..

[42] Li J., Cheng J.-H., Shi J.-Y., Huang F. (2012). Brief introduction of back propagation (BP) neural network algorithm and its improvement. Adv. Comput. Sci. Inf. Eng..

[43] Van den Broeck G., Lykov A., Schleich M., Suciu D. (2022). On the tractability of SHAP explanations. J. Artif. Intell. Res..

[44] Wang M., Wang Y., Islam M., Wang Y., Wang Y., Hwang J., Fan Y. (2026). Dual machine learning pinpoints the Radius of Informative Structural Environments in metallic glasses. npj Comput. Mater..

[45] Zheng Z., Yang D., Zeng L., Mughees N. (2025). A deep learning framework for objective aesthetic evaluation of indoor landscapes using CNN-GNN model. Sci. Rep..

